# Therapeutic Potential of Cysteine and Its Derivatives in Dermatology

**DOI:** 10.3390/molecules31081277

**Published:** 2026-04-13

**Authors:** Joon Yong Choi, Weon-Ju Lee, Yong Chool Boo

**Affiliations:** 1 Department of Biomedical Science, The Graduate School, Kyungpook National University, 680 Gukchaebosang-ro, Jung-gu, Daegu 41944, Republic of Korea; halo134679@knu.ac.kr; 2 BK21 Plus KNU Biomedical Convergence Program, Kyungpook National University, 680 Gukchaebosang-ro, Jung-gu, Daegu 41944, Republic of Korea; 3 Bio-Medical Research Institute, Kyungpook National University Hospital, 135 Dongdeok-ro, Jung-gu, Daegu 41940, Republic of Korea; weonju@knu.ac.kr; 4 Department of Dermatology, School of Medicine, Kyungpook National University, 130 Dongdeok-ro, Jung-gu, Daegu 41944, Republic of Korea; 5 Department of Molecular Medicine, School of Medicine, Kyungpook National University, 680 Gukchaebosang-ro, Jung-gu, Daegu 41944, Republic of Korea; 6 Cell and Matrix Research Institute, Kyungpook National University, 680 Gukchaebosang-ro, Jung-gu, Daegu 41944, Republic of Korea

**Keywords:** cysteine, derivatives, dermatology

## Abstract

Cysteine is a sulfur-containing amino acid that plays a central role in skin physiology through thiol-mediated redox regulation and glutathione (GSH) synthesis. It critically influences melanogenesis, collagen homeostasis, and wound healing. However, its clinical application is limited by poor stability and bioavailability. In this review, we provide a mechanistic and comparative analysis of cysteine and its derivatives, including N-acetylcysteine (NAC), cysteinamide (C-NH_2_), GSH, and related compounds. These derivatives regulate melanogenesis by modulating dopaquinone pathways and tyrosinase activity, maintain collagen balance by preserving redox-sensitive enzymatic processes, and enhance wound healing through antioxidant and anti-inflammatory mechanisms. Importantly, chemical modifications such as acetylation, amidation, and esterification improve pharmacokinetic properties, enabling more effective intracellular delivery. Furthermore, different derivatives exhibit distinct advantages depending on biological context, highlighting the importance of compound selection. Overall, cysteine derivatives emerge as promising therapeutic candidates for dermatological applications, particularly in pigmentation disorders and impaired wound healing. Future studies should focus on in vivo validation and clinical translation.

## 1. Introduction

Skin physiology is maintained through tightly regulated processes including melanogenesis, collagen homeostasis, and wound healing. These processes are highly sensitive to intracellular redox balance, which governs cellular responses to oxidative stress, inflammation, and metabolic dysfunction [1,2,3]. Disruption of redox homeostasis is a common underlying feature in various dermatological conditions, such as hyperpigmentation, skin aging, fibrosis, and impaired wound healing, particularly in diabetic contexts [4,5,6]. Reactive oxygen species (ROS), when excessively accumulated, not only alter melanogenic pathways but also impair collagen synthesis and promote matrix degradation, ultimately leading to structural and functional deterioration of the skin [7,8,9]. Therefore, modulation of redox balance has emerged as a central therapeutic strategy in dermatology [10].

Among endogenous regulators of redox homeostasis, cysteine plays a pivotal role due to its thiol (-SH)-containing structure, which enables direct antioxidant activity and serves as a precursor for glutathione (GSH), the most abundant intracellular antioxidant [11,12]. Through these properties, cysteine contributes to the regulation of melanogenesis by influencing dopaquinone pathways [13,14,15], contributes to the maintenance of collagen homeostasis by regulating redox-sensitive pathways [8,9,16], and is implicated in wound healing processes through modulation of oxidative and inflammatory responses [6,17,18]. Despite its biological importance, the direct application of cysteine as a therapeutic agent is limited by its intrinsic instability, susceptibility to oxidation, and low bioavailability [19,20,21,22]. These limitations hinder its effective delivery and sustained activity in biological systems, restricting its clinical utility.

Accordingly, increasing attention has been directed toward strategies that modulate the redox and cellular microenvironment as a therapeutic approach to overcome such limitations. More broadly, recent advances in regenerative medicine and biomaterials have demonstrated that modulation of oxidative and cellular microenvironments can be achieved through ROS-responsive scaffolds, gradient biomaterial design, stem cell- and cell-reprogramming platforms, organoid-based disease models, and nanoreactor systems. These broader developments support the translational relevance of redox-oriented therapeutic strategies and provide an important conceptual backdrop for the development of cysteine-based interventions in dermatology [23,24,25,26,27].

To overcome these challenges, various cysteine derivatives have been developed through chemical modifications such as acetylation, amidation, and esterification, which enhance molecular stability, membrane permeability, and pharmacokinetic properties. Notably, these derivatives—including N-acetylcysteine (NAC), cysteinamide (C-NH_2_), GSH, and related compounds—exhibit distinct functional advantages depending on biological context, suggesting that derivative selection is a critical factor in therapeutic design [28,29,30]. In this review, we provide a mechanistic and comparative analysis of cysteine and its derivatives in dermatology, focusing on their roles in melanogenesis, collagen metabolism, and wound healing. Furthermore, we highlight their differential properties, therapeutic potential, and limitations to propose a strategic framework for future translational applications.

## 2. Dermatological Relevance of Cysteine

Skin physiology is governed by tightly coordinated biochemical processes, including melanogenesis, collagen homeostasis, and wound healing, all of which are highly dependent on intracellular redox balance [1,31]. ROS function as signaling molecules under physiological conditions but induce cellular dysfunction when excessively accumulated, contributing to pigmentation disorders, skin aging, fibrosis, and impaired wound repair [4,6,32]. As a thiol-containing amino acid, cysteine plays a central role in maintaining redox homeostasis through direct antioxidant activity and as a precursor of GSH, the major intracellular redox buffer [11,33,34]. Through these functions, cysteine integrates multiple regulatory pathways in the skin, acting as a key mediator that links oxidative stress to major dermatological processes [35].

Melanin synthesis begins when the enzyme tyrosinase converts L-tyrosine into L-DOPA and dopaquinone. At this point, the pathway for dopaquinone splits into two directions, and the presence or absence of cysteine is decisive [36,37]. If cysteine is deficient or absent, dopaquinone proceeds along the eumelanin synthesis pathway, producing black–brown pigment. Conversely, when sufficient cysteine is present, dopaquinone binds with cysteine to form cysteinyldopa, which then leads directly to the pheomelanin synthesis pathway [38]. Thus, cysteine redirects the reaction of dopaquinone during melanin synthesis, promoting the production of pheomelanin—a lighter-colored, yellow-red pigment—compared to eumelanin [39,40].

Additionally, cysteine alleviates oxidative stress in melanocytes by eliminating ROS generated during melanin synthesis through its own antioxidant effects. This can indirectly inhibit or regulate tyrosinase activity [41]. Indeed, cysteine and its derivative NAC have been reported to suppress melanin production and exhibit skin-whitening effects [42,43]. Consequently, cysteine induces pheomelanin production by directly binding to dopaquinone during melanin synthesis, while also influencing the activity of melanin synthesis enzymes through its antioxidant action. Thus, cysteine functions as a key factor determining not only the total amount of melanin but also its type (eumelanin to pheomelanin ratio) [44,45].

Cysteine acts as a crucial regulator in maintaining collagen homeostasis. Cysteine is not only an amino acid constituting proteins but also a precursor to GSH, a key metabolic product determining the intracellular redox state [46,47]. Therefore, alterations in cysteine metabolism directly impact collagen synthesis and degradation, and consequently, tissue homeostasis [48].

Cysteine plays two major roles in collagen synthesis. First, GSH derived from cysteine helps maintain the activity of enzymes necessary for procollagen synthesis. The hydroxylation reactions of proline and lysine residues in procollagen are mediated by iron ion (Fe^2+^)-dependent enzymes that are vulnerable to oxidation. GSH stabilizes the reduced state of iron ions, thereby preserving enzyme activity [49,50,51]. Second, at the C-terminus of procollagen, disulfide bonds form between cysteine residues, inducing the correct triple helix structure. Cysteine deficiency impairs these disulfide bond formations, leading to protein folding failure and consequently disrupting extracellular collagen secretion [52,53].

Conversely, cysteine metabolism also plays a crucial role in collagen degradation. Increased oxidative stress promotes the expression of matrix metalloproteinases (MMPs), accelerating collagen breakdown [54,55]. However, cysteine metabolites like GSH and hydrogen sulfide (H_2_S) mitigate this MMP activation by suppressing oxidative stress [54,56]. Furthermore, cathepsins, which directly participate in collagen degradation, are cysteine-dependent proteolytic enzymes [57,58]. Changes in cysteine metabolism also affect the expression and activity of these enzymes [58].

This regulation holds significant importance under both physiological and pathological conditions. In diabetes or aging, reduced cysteine and GSH concentrations coupled with ROS accumulation inhibit collagen synthesis and promote degradation, leading to delayed wound healing and decreased skin elasticity [59,60,61]. Conversely, in diseases like fibrosis, oxidative stress activates transforming growth factor-β (TGF-β) signaling, causing excessive collagen accumulation, so regulating cysteine metabolism can mitigate this pathological collagen deposition [62,63,64]. Therefore, cysteine metabolism transcends simple amino acid metabolism. By regulating the redox environment and enzyme activity, it plays a pivotal role in maintaining the balance between collagen synthesis and degradation—that is, preserving collagen homeostasis [46,65].

In this context, cysteine also aids in wound healing. Wound healing occurs through a sequential series of phases: the inflammatory phase, the proliferative phase, and the remodeling phase [66,67]. This process is closely associated with cell migration and proliferation, extracellular matrix (ECM) synthesis, and redox balance regulation [68]. Through its previously described role in maintaining redox balance, it protects cells from the excessive ROS generated after wounding, thereby preventing the initial inflammatory response from becoming overly prolonged [17,69,70]. Additionally, it influences collagen accumulation and ECM restoration by participating in collagen synthesis and ECM remodeling [65,71,72,73].

Consequently, cysteine performs multifaceted functions in the wound healing process: maintaining redox balance, promoting collagen synthesis, and enhancing tissue regeneration. Therefore, the homeostasis of cysteine metabolism is not only essential for normal wound healing but also represents a critical therapeutic target in pathological conditions where healing is delayed, such as diabetes or aging.

**Figure 1:**
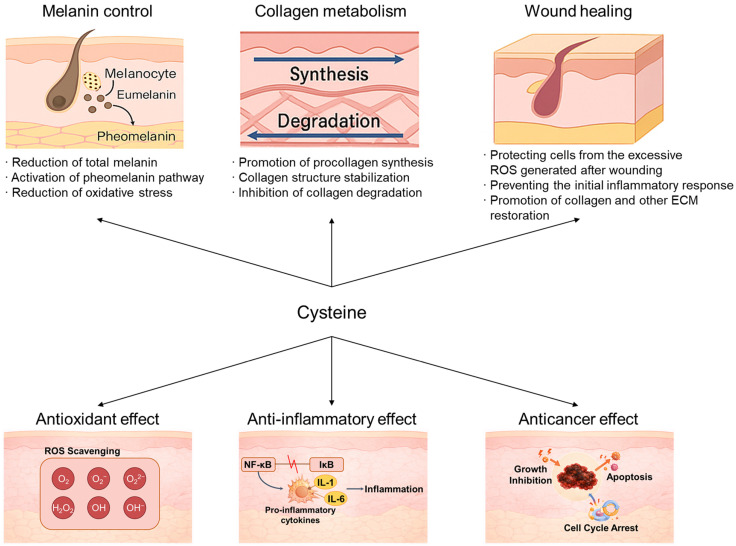
Multifunctional roles of cysteine in skin. Abbreviations: ROS, reactive oxygen species; ECM, extracellular matrix.

Additionally, cysteine has been reported to be involved in antioxidant, anti-inflammatory, and anticancer actions. Cysteine, as a glutathione precursor, increases GSH synthesis, thereby restoring the liver’s antioxidant capacity and enhancing antioxidant-defense capabilities in the high-oxidative-stress environment of diabetes [74]. Cysteine is not merely a ROS scavenger but a key antioxidant amino acid that regulates oxidative stress-based cellular aging processes by converting to GSH and maintaining cellular redox homeostasis [75,76]. Furthermore, cysteine was found to effectively suppress TNF-α-induced inflammatory responses in human coronary arterial endothelial cells (HCAECs), mediated through inhibition of NF-κB activation, IκBα degradation, CD62E expression, and IL-6 production [77]. Moreover, cysteine mitigates cancer progression by suppressing arsenic-mediated cancer promotion [78]. However, contrary to cysteine’s general recognition as an antioxidant protective molecule, excessive cysteine accumulation in cancer cells has been reported to promote the cell cycle alongside increased Cyclin D protein, accelerating cancer cell proliferation. Consequently, some cancer research utilizes an anticancer approach through cysteine-deficient diets (Figure 1) [79,80].

## 3. Limitations of Cysteine for Biological Applications

Despite its central role in redox regulation and skin physiology, the direct application of cysteine as a therapeutic agent is significantly limited by its intrinsic chemical instability and unfavorable physicochemical properties. The thiol group of cysteine is highly reactive and readily undergoes oxidation to form cystine under physiological and experimental conditions, leading to reduced bioavailability and inconsistent biological activity [12,20]. In aqueous environments and in vivo systems, this rapid oxidation impairs the ability to maintain effective intracellular concentrations of cysteine, thereby limiting its pharmacological utility [21]. Furthermore, cysteine exhibits poor membrane permeability due to its polar structure, restricting its efficient transport across lipid bilayers and reducing tissue distribution [81,82]. These limitations collectively hinder the direct use of cysteine in therapeutic formulations.

Beyond its physicochemical instability, cysteine also presents biological and pharmacological limitations that complicate its therapeutic application. The intracellular concentration of cysteine is tightly regulated, and excessive supplementation may disrupt redox homeostasis rather than restore it, potentially leading to adverse cellular effects [34,83]. In particular, while cysteine-derived antioxidants can protect normal cells from oxidative damage, elevated cysteine levels in certain pathological contexts, such as cancer, may enhance tumor cell survival and proliferation by supporting redox adaptation and metabolic activity [84,85]. Additionally, the rapid metabolism and short half-life of cysteine further limit its sustained biological activity, making it difficult to achieve controlled and targeted therapeutic effects [20]. These context-dependent and dose-sensitive effects underscore the challenges associated with using unmodified cysteine as a pharmacological agent.

To overcome these limitations, chemical modification of cysteine has emerged as a strategic approach to enhance its therapeutic potential. Structural modifications such as acetylation, amidation, and esterification can improve oxidative stability, increase lipophilicity, and facilitate cellular uptake, thereby enhancing bioavailability and pharmacokinetic profiles [57,61]. Importantly, these modifications not only address the limitations of native cysteine but also enable functional diversification, allowing specific derivatives to target distinct biological processes more effectively. As a result, cysteine derivatives offer improved stability, controlled delivery, and context-specific activity, making them more suitable candidates for dermatological applications. These advances provide the foundation for the development of next-generation redox-modulating therapeutics, which will be discussed in the following sections.

## 4. Types and Characteristics of Cysteine Derivatives

To overcome the intrinsic limitations of cysteine, a variety of chemically modified derivatives have been developed to improve stability, bioavailability, and functional specificity. These derivatives are designed to modulate key physicochemical properties, including resistance to oxidation, membrane permeability, and intracellular delivery efficiency [57,61]. Importantly, structural modifications such as acetylation, amidation, and esterification not only enhance pharmacokinetic properties but also influence biological activity by altering redox potential and molecular interactions [20,86]. Based on their chemical features and functional characteristics, cysteine derivatives can be broadly categorized into acetylated forms, amide derivatives, esterified compounds, and naturally occurring analogs. This classification provides a framework for understanding their differential roles and therapeutic relevance in dermatology.

**Figure 2:**
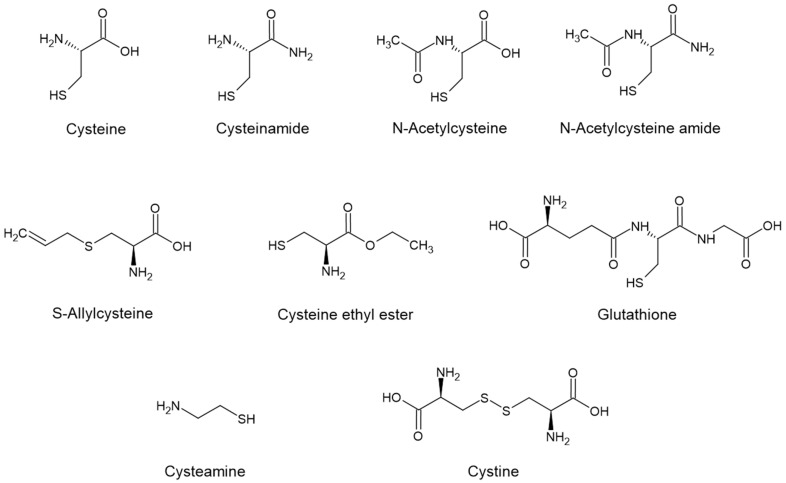
The chemical structures of cysteine (Cys), cysteinamide (C-NH_2_), N-acetylcysteine (NAC), N-acetylcysteine amide (NAC-NH_2_), S-allylcysteine (SAC), cysteine ethyl ester (CEE), glutathione (GSH), cysteamine, and cystine.

The chemical structures of cysteine and its derivatives are shown in Figure 2.

### 4.1. Acetylated Derivatives

Acetylated derivatives, particularly NAC, represent the most widely studied class of cysteine-based compounds. The introduction of an acetyl group reduces the reactivity of the amino group and enhances resistance to oxidation, thereby improving stability compared with native cysteine [57]. NAC is efficiently deacetylated intracellularly to release cysteine, serving as a precursor for GSH synthesis and exerting potent antioxidant effects [87,88]. Due to its favorable pharmacological profile, NAC has been extensively used in clinical settings, including as a mucolytic agent and an antidote for acetaminophen toxicity [89]. In dermatological contexts, NAC contributes to redox regulation, modulation of inflammatory responses, and improvement of wound healing processes [17,28]. However, its relatively limited membrane permeability and dependence on intracellular conversion may restrict its efficiency in certain applications [86].

### 4.2. Amidated Derivatives

Amidated derivatives of cysteine, such as C-NH_2_ and N-acetylcysteine amide (NAC-NH_2_), are designed to enhance lipophilicity and membrane permeability by replacing the carboxyl group with an amide moiety. This modification reduces ionization at physiological pH, facilitating cellular uptake and improving intracellular delivery [90,91]. C-NH_2_ has been reported to exhibit strong inhibitory effects on melanogenesis through direct interaction with dopaquinone and modulation of tyrosinase activity [29], making it particularly relevant for pigmentation control [63]. NAC-NH_2_, on the other hand, demonstrates superior antioxidant capacity and enhanced bioavailability compared with NAC, including improved penetration across biological barriers [88]. These properties suggest that amide derivatives may offer functional advantages in applications requiring efficient intracellular targeting and rapid redox modulation.

### 4.3. Esterified Derivatives

Esterified derivatives, such as cysteine ethyl ester (CEE), are developed to improve membrane permeability and facilitate rapid intracellular delivery of cysteine. The esterification of the carboxyl group increases lipophilicity, allowing these compounds to readily cross lipid bilayers and subsequently undergo intracellular hydrolysis to release cysteine [86,92]. As a result, esterified derivatives serve as effective cysteine donors, enhancing intracellular GSH levels and reducing oxidative stress [93]. These properties make them particularly useful in experimental models of oxidative injury and metabolic dysfunction [33]. However, the rapid hydrolysis and potential variability in esterase activity may influence their pharmacokinetics and limit precise control over cysteine release [86].

### 4.4. Naturally Derived and Other Functional Analogs

In addition to synthetic derivatives, several naturally occurring cysteine-related compounds exhibit significant biological activity. GSH, a tripeptide containing cysteine, is the most abundant intracellular antioxidant and plays a central role in redox homeostasis, detoxification, and cellular signaling [11,12]. S-allylcysteine (SAC), derived from aged garlic extract, is characterized by high stability and bioavailability, and exerts antioxidant and anti-inflammatory effects through mechanisms such as Nrf2 activation [94,95]. Cysteamine, a decarboxylated derivative of cysteine, exhibits strong reducing capacity and has been investigated for its skin-lightening and anti-inflammatory effects, as well as its clinical use in metabolic disorders [96]. Cystine, the oxidized dimer of cysteine, serves as a storage and transport form and contributes to redox buffering in biological systems [82,97]. These compounds highlight the diversity of cysteine-related molecules and their potential functional relevance in dermatology.

Collectively, cysteine derivatives exhibit distinct advantages depending on their chemical structure and biological context. Acetylated derivatives such as NAC provide stability and serve as efficient GSH precursors but may be limited by cellular uptake [20,28,86]. Amide derivatives offer enhanced membrane permeability and stronger intracellular activity, making them suitable for targeting specific cellular processes such as melanogenesis [29,90]. Esterified derivatives function as rapid cysteine donors, enabling efficient modulation of intracellular redox status, although their pharmacokinetics may be less controlled [86,92]. Naturally derived compounds such as GSH, SAC, and cysteamine provide additional functional diversity, including antioxidant, anti-inflammatory, and signaling-related effects [12,94,96]. Therefore, the selection of appropriate cysteine derivatives should be guided by their specific physicochemical properties and targeted biological functions, rather than a uniform application of all compounds. This comparative perspective is essential for the rational design of cysteine-based therapeutic strategies in dermatology.

## 5. Dermatological Effects of Cysteine Derivatives

### 5.1. Effects of Cysteine Derivatives on Melanin Control

Cysteine derivatives play a critical role in regulating melanogenesis through both chemical and redox-dependent mechanisms. A key mechanism involves the interaction of thiol groups with dopaquinone, redirecting the melanogenic pathway from eumelanin toward pheomelanin synthesis, thereby reducing overall pigmentation [14,15,16]. In addition, several derivatives directly modulate tyrosinase activity, the rate-limiting enzyme in melanin synthesis. Melanin plays a crucial role in protecting the skin from ultraviolet radiation, but excessive melanin causes dark pigmentation, making proper regulation essential. In MNT-1 human melanoma cells and normal human epidermal melanocytes (HEMs), C-NH_2_ reduced total melanin production without cytotoxicity, particularly decreasing eumelanin. C-NH_2_ inhibited enzyme activity via TYR-Cu^2+^ chelation and induced the conversion of dopaquinone into a DOPA-C-NH_2_ conjugate, thereby bypassing the dopachrome/eumelanin pathway to suppress eumelanin synthesis [29]. GSH binds to the copper active site of tyrosinase, a key enzyme in melanin synthesis, inhibiting its activity. It also promotes the binding of dopaquinone and cysteine, facilitating the conversion of eumelanin to the lighter-colored pheomelanin. Furthermore, GSH downregulates MITF, which controls tyrosinase expression and melanocyte proliferation [98]. Recent research further suggests that the route of administration critically influences both efficacy and safety, particularly in the case of GSH-based skin-lightening approaches [98,99]. Clinical studies on cysteamine report that daily application of 5% cysteamine cream to melasma lesions for 4 months resulted in greater improvement compared to a placebo group [96]. These findings indicate that cysteine derivatives not only alter melanin synthesis pathways but also regulate enzymatic and transcriptional processes involved in pigmentation.

### 5.2. Effects of Cysteine Derivatives on Collagen Metabolism

Cysteine derivatives contribute to the regulation of collagen metabolism by maintaining the balance between synthesis and degradation through redox control. Collagen constitutes the majority of the skin’s dermis layer and maintains skin elasticity and moisture. Therefore, a decrease in collagen easily leads to skin aging and wrinkles. To prevent this, it is essential to appropriately regulate collagen metabolism. When rat palatal tissue-derived oral mucosal cells are cultured and treated with hydrogen peroxide, cell proliferation and collagen production increase; however, NAC inhibits this increase [100]. In rat cardiac fibroblasts (CFs), NAC suppressed Angiotensin II-induced CF proliferation and collagen synthesis by inhibiting the activation of the NF-kB signaling pathway [101]. SAC, one of the major compounds in aged garlic extract, reduced the mRNA expression of inflammatory and fibrogenic cytokines, including interleukin 6, interferon γ, tumor necrosis factor α, and TGF-β, and also reduced mRNA expression of liver fibrosis biomarkers, including α-smooth muscle actin, fibronectin, and collagen I [102]. Furthermore, SAC reduced mRNA expression of fibrosis genes such as alpha smooth muscle actin (a-SMA), fibronectin, collagen I, and collagen III, as well as a-SMA protein levels in bleomycin (BLM)-induced pulmonary fibrosis in mice [103]. TGF-β depletes GSH in fibroblasts, increasing collagen I expression and accumulation. Supplementing GSH inhibits TGF-β-induced collagen accumulation and normalizes collagen degradation [104]. These findings highlight the dual role of cysteine derivatives in preventing both collagen deficiency and excessive fibrosis, depending on the pathological context.

### 5.3. Effects of Cysteine Derivatives on Wound Healing

Cysteine derivatives have demonstrated significant potential in promoting wound healing by modulating oxidative stress, inflammation, and tissue regeneration. The wound healing process is a sequence of various activities related to tissue restoration. When a skin wound was created in Wistar rats and treated with 3% NAC cream for 21 days, increased angiogenesis and wound healing rates were observed compared to the control group [105]. In db/db mice, a type 2 diabetes model, treatment with a hydrogel containing 5% NAC resulted in increased skin proliferation area and improved wound closure rate compared to the control group [106]. In a rat ischemic wound model, topical application of esterified GSH was confirmed to enhance wound healing by reducing oxidative stress through increased intracellular GSH, preventing keratinocyte apoptosis, and increasing fibroblast contractile capacity [107]. Importantly, these findings suggest that cysteine-based compounds may be particularly beneficial in pathological conditions characterized by impaired healing and chronic oxidative stress [108].

### 5.4. Antioxidant Effects of Cysteine Derivatives

The antioxidant activity of cysteine derivatives represents their most fundamental and widely recognized biological function. NAC acts as a precursor to GSH, promoting GSH biosynthesis and functioning as an antioxidant that scavenges free oxygen radicals. It is used as a therapeutic agent for certain conditions such as acetaminophen toxicity, chronic bronchitis, ulcerative colitis, liver cancer, hemodialysis, and asthma [20,109]. NAC-NH_2_, the amide form of NAC, exhibits high lipophilicity, resulting in superior cellular permeability and BBB penetration. This characteristic enables enhanced efficacy in GSH supplementation and ROS scavenging, leading to high bioavailability [110,111]. SAC possesses multiple antioxidant mechanisms, including free radical scavenging, antioxidant enzyme induction, Nrf2 factor activation, and prooxidant enzyme inhibition [94]. As a natural antioxidant with protective effects against cerebral ischemia or cancer, it reduced ROS generated under hypoxia induced by cobalt chloride (CoCl_2_) and protected cells from cell death [112]. CEE, a cell membrane-permeable carboxylate ester, rapidly enters tissues or the brain, where it is broken down by carboxylesterase to increase cysteine levels. This elevated cysteine promotes GSH synthesis. It also reduced oxidative stress and improved gas exchange abnormalities in opioid analgesic models, such as morphine and fentanyl [113]. GSH is an antioxidant involved in the primary cellular reduction system, eliminating ROS, regulating protein S-glutathionylation, and activating the Nrf2 antioxidant pathway to reduce oxidative damage [114]. Cysteamine demonstrated radiation protection and potent antioxidant properties when tissue was irradiated, confirming its efficacy as a radioprotective agent and antioxidant [115]. Collectively, these properties underscore the central role of cysteine derivatives in maintaining redox homeostasis and protecting cells from oxidative injury.

### 5.5. Anti-Inflammatory Effects of Cysteine Derivatives

Cysteine derivatives exert significant anti-inflammatory effects by modulating redox-sensitive signaling pathways. Oxidative stress is closely linked to the activation of inflammatory cascades, particularly through transcription factors such as NF-κB. In Pam212 murine keratinocytes, 2-hydroxyethyl methacrylate (HEMA) induced IL-1α secretion and cytotoxicity, which was associated with increased ROS production and elevated calpain enzyme activity. NAC inhibited HEMA-induced IL-1α secretion, ROS production, and calpain activity. Furthermore, NAC suppressed HEMA-induced increases in IL-1α secretion in IL-1 KO mice [116]. NAC exhibited anti-inflammatory effects by suppressing the secretion of inflammatory factors such as tumor necrosis factor alpha (TNF-α) and interleukins (IL-6 and IL-1) in LPS-treated macrophages through the inhibition of nuclear factor kappa B (NF-κB) activity [117,118]. SAC suppressed TNF-α-induced inflammatory cytokine expression in HaCaT keratinocytes. SAC inhibited TNF-α-induced activation of the NF-κB pathway and continuously activated the ERK pathway [119]. Furthermore, in a renal inflammation model of diabetic mice, SAC administration suppressed NF-κB and significantly reduced the expression of ROS, IL-6, TNF-α, and prostaglandin E2 [120]. In lipopolysaccharide (LPS)-stimulated murine RAW 264.7 macrophages and human macrophages, N-butanoyl GSH (GSH-C4) reduced the expression of pro-inflammatory cytokines such as IL-1β, IL-6, and TNF-α [121]. Cysteamine was confirmed to mitigate excessive immune responses by regulating inflammatory cytokines such as IFN-γ, TNF, and IL-2 [122]. These findings indicate that the anti-inflammatory effects of cysteine derivatives are closely linked to their antioxidant properties and play an important role in dermatological conditions characterized by persistent inflammation.

### 5.6. Anticancer Effects of Cysteine Derivatives

The role of cysteine derivatives in cancer biology is complex and context-dependent, reflecting the dual nature of redox regulation in tumor progression. NAC protected mouse melanocytes (melan-a cells) from UV-induced DNA lesions such as 8-oxoguanine (8-OG) induced by UV radiation and the depletion of free reduced thiols. In mice with induced melanoma, NAC administration reduced thiol depletion, inhibited 8-OG formation in the skin, and significantly delayed UV-induced melanocyte tumors compared to the control group [42]. ROS such as peroxides and hydrogen peroxide stimulate tumor cells and immune cells. In this regard, NAC could be utilized as an adjuvant to anticancer drugs by regulating oxidative stress and inflammatory responses in precancerous stages or tumor microenvironment metabolism. However, excessive antioxidant activity may adversely affect tumor immunity or ROS-dependent cell death, necessitating optimization [123]. Radiation causes microvascular damage and oxidative stress, damaging salivary glands. In bovine aortic endothelial cells (BAECs), NAC-NH_2_ reduced radiation-induced endothelial cell death. Furthermore, in a C3H mouse model, pre-treatment with NAC-NH_2_ before head and neck irradiation protected against radiation-induced salivary gland dysfunction and reduced microvascular loss, suggesting potential of NAC-NH_2_ as a radioprotective agent [124]. In bladder cancer cell lines, SAC inhibited cell proliferation and colony formation, induced apoptosis, and arrested the cell cycle at the S phase, thereby preventing bladder cancer cell growth [125]. In the human epithelial ovarian cancer cell line A2780, SAC also induced G1/S phase arrest and apoptosis, reduced cell migration, and decreased the expression of proteins involved in proliferation and metastasis, such as Wnt5a, p-AKT, and c-Jun [126]. In cases of cellular damage, reactive oxygen species (ROS) production increases. Excessive ROS production can lead to various diseases, including cancer. GSH can prevent this by maintaining redox homeostasis and can also serve as a useful protective factor by mitigating the toxicity of anticancer or radiation therapy [127]. Cysteamine, an amino thiol molecule produced by cells during the degradation of coenzyme A, is used in the treatment of various conditions such as cystinosis and neurodegenerative diseases. It has shown improved efficacy in the treatment of multiple cancers including gastrointestinal cancer, pancreatic cancer, sarcomas, hepatocellular carcinoma, and melanoma [128]. It has been suggested that cysteamine can be utilized in cancer treatment by inhibiting MMP activity in glioblastoma (GBM) cells, thereby suppressing tumor cell invasion and metastasis [129]. These findings highlight the need to carefully consider dose, context, and disease stage when applying cysteine derivatives in therapeutic strategies, particularly in oncology-related dermatological conditions.

Selected experimental and clinical studies on the dermatological effects of cysteine derivatives are summarized in Table 1.

## 6. Discussion

Cysteine plays a central role in skin physiology as a thiol-containing amino acid that governs redox homeostasis, GSH synthesis, and disulfide bond formation [11,12]. Through these mechanisms, cysteine is intricately involved in the regulation of melanogenesis, maintenance of collagen homeostasis, and promotion of wound healing [4,17]. However, despite its biological importance, the direct therapeutic application of native cysteine remains limited due to its intrinsic instability, rapid oxidation, poor membrane permeability, and short biological half-life [20,86]. These physicochemical and pharmacological constraints significantly restrict its clinical utility.

**Figure 3:**
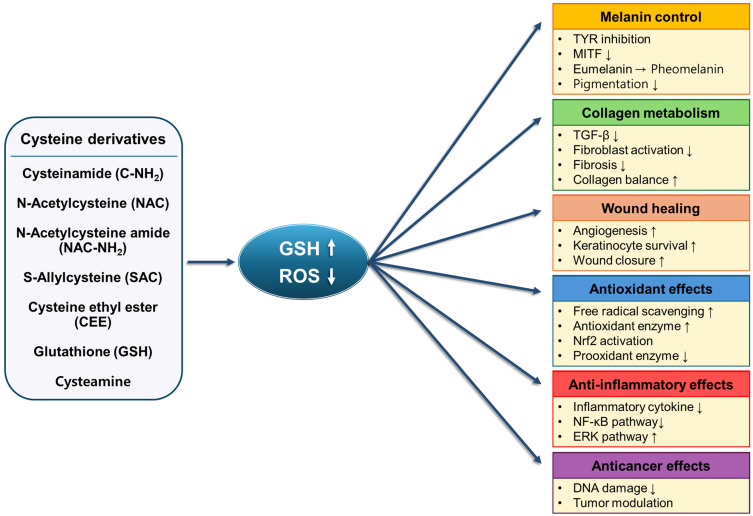
Integrated mechanisms of cysteine derivatives in dermatological effects. Abbreviations: ROS, reactive oxygen species; TYR, tyrosinase; MITF, microphthalmia-associated transcription factor; TGF-β, transforming growth factor-β; Nrf2, nuclear factor erythroid-2-related factor 2; NF-κB, nuclear factor kappa-light-chain-enhancer of activated B cells; ERK, extracellular signal-regulated kinase. The symbol ↑ and ↓ denote increase and decrease, respectively.

To address these limitations, a wide range of cysteine derivatives has been developed through chemical modifications such as acetylation, amidation, and esterification. These modifications enhance molecular stability, improve cellular uptake, and enable more efficient intracellular delivery [20,86,90]. Importantly, different derivatives exhibit distinct functional advantages depending on their structural properties and biological context [28,30]. For example, acetylated derivatives such as NAC primarily act as GSH precursors and antioxidants [88], while amide derivatives demonstrate enhanced membrane permeability and stronger intracellular activity [29,90], and esterified forms function as efficient cysteine donors [86,92]. In addition, naturally derived compounds such as GSH, SAC, and cysteamine further expand the functional diversity of cysteine-related molecules by providing antioxidant, anti-inflammatory, and signaling-related effects (Figure 3) [12,94,96,130].

Collectively, these findings highlight that cysteine derivatives are not merely substitutes for native cysteine but represent a strategically optimized class of redox-active compounds with improved pharmacological properties. Their ability to modulate key dermatological processes—including pigmentation, extracellular matrix remodeling, and tissue regeneration—positions them as promising candidates for therapeutic applications in hyperpigmentation disorders, skin aging, fibrosis, and chronic wounds such as diabetic ulcers. However, despite encouraging preclinical evidence, several challenges remain, including limited clinical validation, insufficient comparative studies between derivatives, and a lack of standardized criteria for compound selection based on specific pathological conditions.

Future research should focus on systematic comparative evaluations of different cysteine derivatives, with particular attention to their pharmacokinetics, tissue specificity, and long-term safety. In addition, well-designed in vivo and clinical studies are required to validate their therapeutic efficacy and to establish optimal dosing strategies. Furthermore, the integration of cysteine-based compounds into advanced delivery systems, such as nanocarriers or hydrogel platforms, may further enhance their clinical applicability. Ultimately, a deeper understanding of thiol-based redox regulation and its interaction with disease-specific pathways will be essential for the rational design of next-generation dermatological therapeutics based on cysteine derivatives.

## Figures and Tables

**Table 1:** Experimental and clinical studies on the dermatological effects of cysteine derivatives.

Dermatological Effects	Experimental Models	Evidence Level	Inducing Factors	Cysteine Derivatives	Outcomes	Primary Mechanism	Clinical Relevance	Limitations	Ref.
Melanin control	MNT-1 melanoma cells, human epidermal melanocytes (HEMs)	in vitro		C-NH_2_	Total melanin ↓, eumelanin ↓	Tyrosinase inhibition via TYR-Cu^2+^ chelation, pheomelanin pathway via DOPA-cysteinamide conjugate	Low (Only basic research)	Only in vitro evidence	[29]
	Patients with melasma, melanocytes	in vitro, clinical		GSH	Total melanin ↓, eumelanin ↓, MITF expression ↓	Tyrosinase inhibition via TYR-Cu^2+^ chelation, pheomelanin pathway via DOPA-cysteine conjugate	High (Products available)		[98]
	Patients with facial melasma	clinical		Cysteamine	Melasma improvement		High	Lack of histological assessments and assessment of systemic absorption	[96]
Collagen metabolism	Rat palatal tissue derived oral mucosal cells	in vitro	Hydrogen peroxide (H_2_O_2_)	NAC	Cell proliferation ↓, collagen production ↓		Moderate (Preclinical research)	Research into the mechanism is required	[100]
	Sprague-Dawley rats	in vivo	Angiotensin II	NAC	Fibrosis area ↓, collagen I ↓, GPX1, GPX3, SOD1, and SOD 2 expression ↑, ROS generation ↓	Increased expression of antioxidant-related genes			[101]
	Rat cardiac fibroblasts (CFs)	in vitro			Proliferation ↓, collagen synthesis ↓	Inhibition of the NF-κB pathway			
	Sprague-Dawley rats	in vivo		SAC	mRNA expression of inflammatory (IL-6, IFN-γ, TNF-α), fibrogenic (TGF-β) cytokines and liver fibrosis biomarkers (α-SMA, fibronectin, collagen I) ↓		Moderate	Further clinical application is required	[102]
	Pulmonary fibrosis induced C57BL/6 mice	in vivo	Bleomycin (BLM)	SAC	mRNA expression of fibrosis genes (α-SMA, fibronectin, collagen I, and collagen III) ↓, α-SMA protein level ↓				[103]
	NIH 3T3 murine embryo fibroblasts	in vitro	TGF-β	GSH	Collagen accumulation ↓, normalization of collagen degradation		Low	Further clinical application is required	[104]
Wound healing	Wistar rats	in vivo		NAC	Angiogenesis ↑, wound healing rate ↑		Moderate	Clinical trials are needed to assess the use of NAC	[105]
	db/db mice	in vivo		NAC	Skin proliferation ↑, wound closure ↑				[106]
	Sprague-Dawley rats	in vivo		Esterified GSH	Wound healing ↑, TIMP-1 level ↑	Reduction in oxidative stress	Moderate	Clinical trials involving topical application are required	[107]
Antioxidant effects	PC12 cells	in vitro	Cobalt chloride (CoCl_2_)	SAC	ROS generation ↓, cell toxicity ↓		Low	Research into the mechanism is required	[112]
	Sprague-Dawley rats	in vivo		CEE	GSH synthesis ↑, oxidative stress ↓, improvement of gas exchange abnormality	Cellular permeability via carboxylate ester, cysteine supplementation	Moderate	Research into the mechanism is required	[113]
Anti-inflammatory effects	Pam212 murine keratinocytes	in vitro	2-hydroxyethyl methacrylate (HEMA)	NAC	Inhibition of HEMA-induced IL-1α release, inhibition of intracellular calpain activity and ROS production		Moderate	Inconsistent clinical outcomes	[116]
	IL-1 KO BALB/c mice	in vivo			Inhibition of IL-1α release				
	HaCaT keratinocytes	in vitro	TNF-α	SAC	Inhibition of the NF-κB pathway, activation of the ERK pathway		Moderate	Limited clinical dermatology data	[119]
Anticancer effects	Melan-a mouse melanocytes	in vitro	Irradiation	NAC	Protection from the production of intracellular peroxide, formation of DNA lesions such as 8-oxoguanine (8-OG) ↓, depletion of free reduced thiol ↓		Moderate	Limited clinical dermatology data	[42]
	Hepatocyte growth factor (HGF)/Survivin-Tg mice	in vivo			Formation of DNA lesion 8-OG ↓, depletion of free reduced thiol in skin ↓, delay of the onset of UV-induced melanocytic tumors				
	B16 melanoma cells implanted mice	in vivo		Cysteamine	Reduction in tumor size through combined treatment with doxorubicin		Moderate	Limited clinical dermatology data	[128]

Abbreviations: HEMs, human epidermal melanocytes; C-NH_2_, cysteinamide; TYR, tyrosinase; DOPA, 3,4-dihydroxyphenylalanine; GSH, glutathione; MITF, microphthalmia-associated transcription factor; H_2_O_2_, hydrogen peroxide; NAC, N-acetylcysteine; UV, ultraviolet; 8-OG, 8-oxoguanine; CFs, cardiac fibroblasts; NF-κB, nuclear factor kappa-light-chain-enhancer of activated B cells; SAC, S-allylcysteine; IL-6, interleukin-6; IFN-γ, interferon gamma; TNF-α, tumor necrosis factor α; TGF-β, transforming growth factor-β; α-SMA, alpha smooth muscle actin; BLM, bleomycin; TIMP-1, tissue inhibitor of metalloproteinase 1; CoCl_2_, cobalt chloride; ROS, reactive oxygen species; CEE, cysteine ethyl ester; HEMA, 2-hydroxyethyl methacrylate; ERK, extracellular signal-regulated kinase; 8-OG, 8-oxoguanine; HGF, hepatocyte growth factor; Tg, transgenic. The symbol ↑ and ↓ denote increase and decrease, respectively.

## Data Availability

The original contributions presented in the study are included in the article, and further inquiries can be directed to the corresponding author.
